# Lingual electrotactile discrimination ability is associated with the presence of specific connective tissue structures (papillae) on the tongue surface

**DOI:** 10.1371/journal.pone.0237142

**Published:** 2020-08-07

**Authors:** Tyler S. Allison, Joel Moritz, Philip Turk, Leslie M. Stone-Roy

**Affiliations:** 1 Department of Biomedical Sciences, Colorado State University, Fort Collins, Colorado, United States of America; 2 Department of Mechanical Engineering, Colorado State University, Fort Collins, Colorado, United States of America; 3 Sapien LLC, Fort Collins, Colorado, United States of America; 4 Department of Statistics, Colorado State University, Fort Collins, Colorado, United States of America; Erasmus MC, NETHERLANDS

## Abstract

Electrical stimulation of nerve endings in the tongue can be used to communicate information to users and has been shown to be highly effective in sensory substitution applications. The anterior tip of the tongue has very small somatosensory receptive fields, comparable to those of the finger tips, allowing for precise two-point discrimination and high tactile sensitivity. However, perception of electrotactile stimuli varies significantly between users, and across the tongue surface. Despite this, previous studies all used uniform electrode grids to stimulate a region of the dorsal-medial tongue surface. In an effort to customize electrode layouts for individual users, and thus improve efficacy for sensory substitution applications, we investigated whether specific neuroanatomical and physiological features of the tongue are associated with enhanced ability to perceive active electrodes. Specifically, the study described here was designed to test whether fungiform papillae density and/or propylthiouracil sensitivity are positively or negatively associated with perceived intensity and/or discrimination ability for lingual electrotactile stimuli. Fungiform papillae number and distribution were determined for 15 participants and they were exposed to patterns of electrotactile stimulation (ETS) and asked to report perceived intensity and perceived number of stimuli. Fungiform papillae number and distribution were then compared to ETS characteristics using comprehensive and rigorous statistical analyses. Our results indicate that fungiform papillae density is correlated with enhanced discrimination ability for electrical stimuli. In contrast, papillae density, on average, is not correlated with perceived intensity of active electrodes. However, results for at least one participant suggest that further research is warranted. Our data indicate that propylthiouracil taster status is not related to ETS perceived intensity or discrimination ability. These data indicate that individuals with higher fungiform papillae number and density in the anterior medial tongue region may be better able to use lingual ETS for sensory substitution.

## Introduction

Sensory systems are crucial for providing individuals with information about both the internal and external environments, and are needed to assess and respond to stimuli. Because of their central role in providing the brain with information, damage to a sensory system can be devastating. Assistive devices and aids such as eye glasses, hearing aids, and implants significantly improve the quality of life for many people with sensory loss. Unfortunately, these aids are ineffective in certain cases. Sensory substitution is a promising type of assistive technology that involves transforming stimuli from one modality to another (e.g., sound to touch), enabling a non-functional sensory system to be bypassed and presenting the information to a different, healthy sensory system. Braille, a well-known example of this approach uses touch stimuli to provide information to the brain that would otherwise be transmitted by the visual system. Modern research focuses on the development and testing of specific devices for sensory substitution in an effort to expand available resources for those with sensory impairment [1, 2]. Bach-y-Rita was among the first to suggest that the adult brain is plastic enough for sensory cortices to decode neural impulses from an alternate sensory modality when a primary modality has been damaged. He demonstrated this notion through experiments in which blindfolded subjects were able to discriminate differences in object form when visual information from a camera was translated into vibrational patterns applied to the skin of the subject’s back [3, 4]. While the underlying mechanism is still controversial, there have been numerous studies over the past decade supporting this idea of cross-modal brain plasticity between the somatosensory and visual cortical areas [5–11], as well as between the somatosensory and auditory systems at both the cortical and brainstem levels [12–19]. Since Bach-y-Rita’s initial development of his vision substitution device, multiple groups have worked on improving sensory substitution devices (SSDs) to allow for practical use beyond the laboratory. While many of these devices focus on cutaneous tactile stimulation of the back or hand [20–28], electrotactile stimulation (ETS) of the tongue has certain advantages. The tongue is densely innervated and has a large region of sensory cortex dedicated to processing somatosensory information, it is in a protected environment allowing sensory receptors to terminate close to the surface, and is coated in saliva, an electrolytic solution that provides excellent conductive properties [29–31]. In addition, the somatosensory receptors of the tongue have small receptive fields that result in superior two-point discrimination ability compared to other regions such as the back [20, 32–36]. The tongue also has a lower intensity threshold to ETS relative to the fingertips and can be effectively stimulated with low voltage signals [29]. Several groups have developed successful SSDs involving ETS of the tongue and studies indicate user benefits for people with vestibular impairments [37–46]. In addition to vestibular biofeedback, other lingual SSD biofeedback applications include attempts to improve white cane navigation for visually impaired individuals by providing obstacle distance information [47]. Other vision substitution applications also appear promising [48–51] and additional applications include improving typing efficiency for people with upper limb mobility impairments [52], and even providing guidance during surgeries [53].

The tongue is highly innervated by multiple types of nerve fibers including gustatory, somatosensory, autonomic, and motor [54–56]. The lingual electrotactile techniques used by our research group primarily affect mechanosensitive somatosensory fibers [35], which can be classified by their responses to specific stimuli (mechanical, temperature or noxious). The fibers can be further classified based on peripheral structures, adaptation rate, receptive field, axon diameter, and depth in the tissue (superficial or deep) [32, 33]. Lingual ETS stimulation used in our research is perceived as a brief, tingling sensation similar to that produced by carbonation [29]. Based on this description, it is likely that ETS primarily affects superficial, rapidly adapting, low threshold mechanoreceptors (RA-LTMR) [32, 35, 57]. ETS devices target the anterior portion of the tongue, and the exact distribution of RA-LTMR fibers in this region is unclear. Anatomical studies using rodent models indicate that somatosensory fibers from the lingual nerve innervate lingual papillae, as well as the surrounding epithelium [58–62]. Papillae in the tongue are structures that contain epithelial and connective tissues as well as neuronal endings. Fungiform papillae (FP) are present in the anterior tongue and contain taste buds, but filiform papillae in this region lack taste buds. RA-LTMR fibers are a subset of the lingual nerve somatosensory population and it is not clear if these ETS responsive fibers are uniformly distributed within the anterior lingual epithelium, or more concentrated within, or between papillae. Determining the distribution of fibers relative to fungiform papillae could be valuable since FP distribution and density vary widely between individuals [63, 64]. If ETS sensitive fibers are more prevalent in fungiform papillae for example, people with more FP would be expected to perceive ETS more easily. Previous studies suggest that there might be a correlation between FP density and tactile perception [65, 66]. Unfortunately, these studies focused on FP densities within a single region of the anterior tongue and measured sensitivity thresholds through the identification of embossed letters, which limits the applicability to ETS applications. One report indicated that mild electrical stimulation resulting in sour-metallic taste perception appeared to be more effective in regions with higher fungiform papillae, but this study did not address tactile perception [67]. In addition to differences in fungiform papillae density and distribution, people differ in their ability to taste the bitter compound 6-n-propylthiouracil (PROP) [63, 68]. Although this perceptual characteristic is based on differences in a taste receptor protein, T2R38, there is evidence that PROP sensitivity is related to perception of mechanical and chemical stimuli on the tongue [65, 69, 70].

Although sensory substitution devices using lingual ETS show promise, the somatosensory properties of the tongue vary significantly both within and between individuals [35, 71–74]. While some studies included adjustment of tactile stimulation during studies based on regional differences in perceived intensity [72], there has been no attempt to design lingual SSDs to optimize activation of somatosensory fibers based on regional and individual variability with respect to discriminatory ability as well as perceived intensity. The overall goal of the present study was to provide information that will help optimize tongue stimulation devices based on the neuroanatomy of the tongue. To do this, we tested whether increased fungiform papillae was correlated with increased sensitivity for ETS and whether individuals who were able to detect propylthiouracil (PROP) were better able to perceive active electrodes. These general questions were more specifically addressed by testing the following hypotheses: 1. Individuals with more fungiform papillae will perceive ETS as more intense and have better two-point discrimination ability. 2. For individual participants, ETS in tongue regions with more fungiform papillae will result in better perception than in regions with fewer papillae. 3. Individuals who can detect propylthiouracil will perceive active electrodes as more intense and have better two-point discrimination ability. To test these hypotheses, we recruited 15 subjects and used a custom-designed tongue stimulation device to gather data from participants. These data were then used to generate maps of perceived intensity and discrimination ability as described previously [35]. In addition, we counted each participant’s fungiform papillae in the tested area using a published protocol [75] and tested the PROP sensitivity of each individual. Results from these experiments were then compared and analyzed to determine the relationships between perception of active lingual electrodes and lingual neuroanatomical and physiological features.

## Materials and methods

### Subjects

Fifteen healthy adults, 6 males and 9 females, volunteered to take part in these studies. Subjects were 20–25 years of age and recruited from the university. Prior to participating in the study, subjects were screened using a questionnaire to help eliminate those with oral injuries, infections, or metallic-based devices that might interfere with the studies. Approved subjects went through an informed consent process in accordance with the Declaration of Helsinki. Participant codes were assigned to each individual to maintain confidentiality. For each subject, completion of the experiments took approximately 1 ½ hours and subjects were offered compensation at $8.50/hour for their participation, although some declined and volunteered their time. All procedures and forms were approved by the Institutional Review Board at Colorado State University.

### Tongue stimulation device and mouthpiece arrays

**Fig 1:**
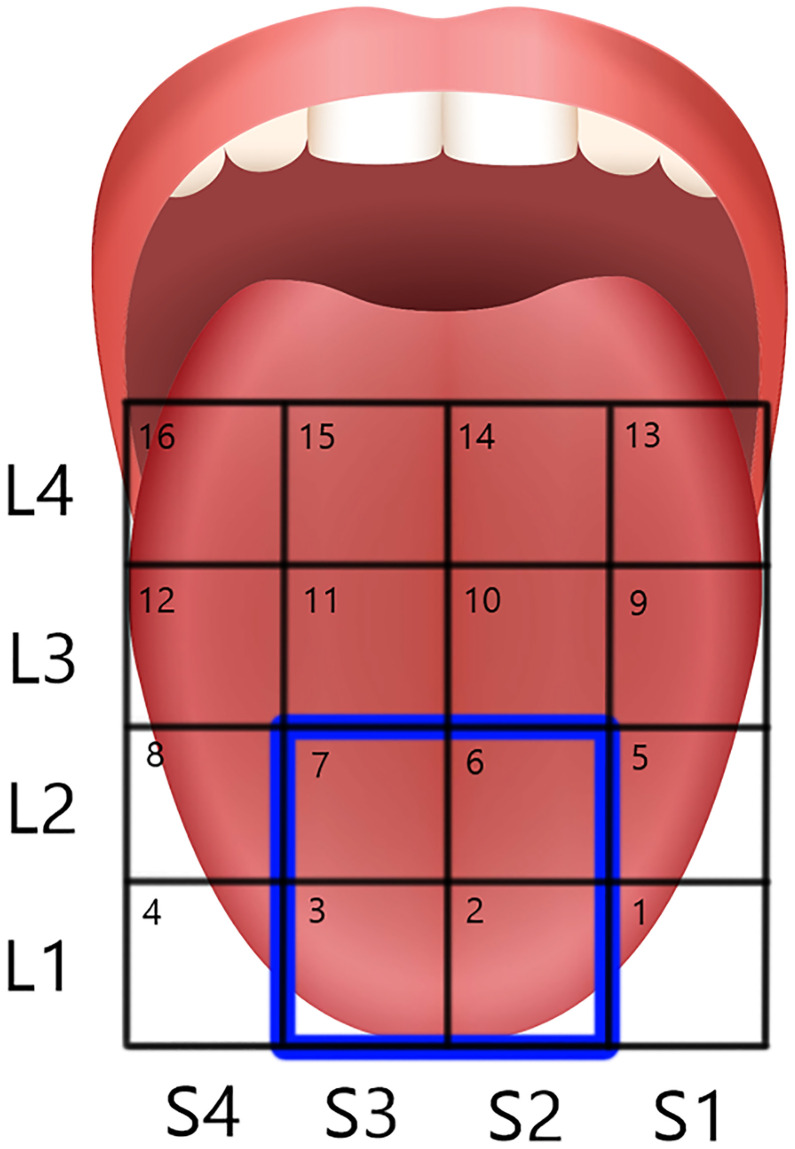
Diagram illustrating the regions of the tongue tested. Locations (L1 –L4) and Subarrays (S1–S4) were identified on tongue photographs by applying a 4 cm^2^ grid overly with Photoshop software. The blue box shows the regions of the tongue that were analyzed in this study. These regions were chosen because they demonstrated the highest sensitivity and discrimination ability. In addition, the tongue surface was the most likely to maintain contact with the electrode array during testing in these regions, thus reducing a potential source of error.

The tongue stimulation device, “The Cthulhu” was designed and constructed as described previously [35, 76] and based on previously published designs [50]. The device was assembled by Sapien, LLC (Fort Collins, CO, USA) using supplies purchased from Mouser Electronics (Mansfield, TX, USA). The ETS device was connected to a mouthpiece containing an electrode array as described previously [35]. Briefly, the array measured 1 cm by 4 cm and consisted of 100 gold-plated electrodes arranged in a 5 x 20 rectangular array, with 2 mm of center-to-center spacing between each electrode. This array was located at one end of a rectangular mouthpiece, with the opposite end attached to the output cables of the Cthulhu device via card-slot connectors. The mouthpiece contained several pairs of holes indexed 1 cm apart, which were used for attaching a plastic stop pad to guide positioning of the mouthpiece array at four different locations along the anterior to posterior axis of the tongue. This allowed data to be collected from a combined 4 cm x 4 cm region of the anterior tongue. Each 1 cm^2^ region was defined by Location (anterior cm = Location 1, most posterior cm tested–Location 4) and by Subarray (left cm = Subarray 1 and right cm = Subarray 4) Fig 1. Data were collected for the entire 4 cm^2^ region, but only data from Location 1 & 2, Subarrays 2 & 3 were used for analyses. Each participant was given their own mouthpiece and stop pad to help prevent disease transmission. Mouthpieces were sterilized prior to initial use by washing with dish detergent, rinsing with distilled water, submerging in non-chlorine bleach for 100 sec, rinsing again with distilled water, and placing the mouthpiece in boiling water for 100 sec. Stop pads were sterilized as part of the manufacturing process since they were extruded from an FDM style 3D-printer at 230°C onto a 110°C platform. Sterile procedures were used to place the mouthpieces and stop pads into individual petri dishes which were wrapped in Parafilm and stored at room temperature. Between experiments, assigned mouthpieces and stop pads were washed with Liquinox, rinsed with distilled water, and dried in a toaster oven using a warm setting. Each participant stored their mouthpiece in a sterile petri dish between testing sessions.

### Somatosensory testing via electrotactile stimulation

Somatosensory testing was done as described previously [35]. Individual electrotactile stimulation experiments typically lasted one hour and consisted of 4 segments corresponding to the 4 anterior-to-posterior 1 cm sections tested (Locations). Subjects were allowed to take a break between segments. Experiments were set up with the participant’s designated, sterile mouthpiece attached to the Cthulhu cables, and the Cthulhu connected to a computer running an open source Arduino serial terminal. An investigator operated the Cthulhu, assisted with proper placement of the mouthpiece, and collected the data. Subjects were asked to place the mouthpiece into their mouths, with the stop pad between their lower lip and teeth, the tip of their tongue pressed against the back of the lower incisors, and the dorsal surface of the tongue pressed against the electrode array. The plastic stop pad was initially placed at the most anterior position to stimulate the most anterior 1 cm of the tongue and then moved back sequentially to test each of the three remaining 1 cm regions of tongue as needed.

Prior to collecting data, a strong, but comfortable setting was determined for each participant by stimulating the anterior tongue as previously described [35]. This setting was used for the remaining experiments involving that subject. This ensured that subject reports involving perceived intensity levels would reflect changes in the subject’s somatosensory ability. After determining this setting, stimuli were presented to the subject by the investigator using the commands entered in the serial terminal. As described previously (2017), stimuli were presented in 116 separate, random sequences for each of the four locations, with 96 of these sequences consisting of two active electrodes spaced 2, 4, 6, or 8 mm apart, in either a horizontal or vertical orientation. The remaining sequences were 20 random, single active electrodes. With each presented sequence, subjects were asked to record how many electrodes they felt (0, 1, 2, or more) and the perceived intensity on a scale from 0 to 10 (with 0 corresponding to a lack of sensation, and 10 being a very strong sensation). Using a hand signal when ready, the investigator would then present the next stimulation. Following completion of each 1 cm location, the subject was asked to move the stop pad to the next position to begin data collection for the corresponding location on the tongue. Data were entered into a previously prepared Excel spreadsheet which cross-referenced the participant responses to each sequence with the list of electrodes activated during that sequence, their location within the array, and the distance between the electrodes. This allowed two-point discrimination to be measured by identifying the spacing of electrodes in sequences where the subject correctly perceived two active electrodes.

### Tongue staining and photography

Diluted blue food dye was used to visualize fungiform papillae according to established procedures [65, 75]. Subjects were asked to avoid food and beverages other than water for one hour prior to the experiment and the mouth was rinsed with water, the tongue was dried with a piece of Whatman’s No. 1 filter paper and diluted blue food dye (Kroger Food Colors) was applied to the tongue using another piece of filter paper saturated in dye. The concentration of dye varied between participants and was determined by applying a few different concentrations and then choosing the dilution that resulted in the maximum contrast between fungiform papillae and the surrounding epithelium. The goal was to have blue stained epithelium and filiform papillae with lighter, pink fungiform papillae. For most participants, the ideal dye dilution was 1:10. For the final staining, the filter paper saturated with diluted blue dye was applied to the subject’s tongue to evenly coat the dorsal surface, as well as the anterior and lateral edges. A second piece of filter paper was used to lightly dry the tongue after dye application. A previously measured and cut 10 mm piece of filter paper was placed on one side of the anterior tongue for a scale. To stabilize the head and tongue for photography, the subject placed their chin and forehead on a Multi-Mount U-Frame Economy Chinrest (model # CRCS-UF-SP-N100-TTB, Miles Research: http://milesresearch.com/pdf/Chinrest-Models.pdf) with the stained tongue protruding between the lips. An Olympus 24x Wide Optical Zoom, Full HD camera was attached to the camera mount 19 cm from the chinrest. Multiple photographs were taken of each participant’s tongue using iAuto camera settings and a 5.5x zoom. The 10 mm strip of filter paper was then moved to the opposite side of the tongue for a second round of photographs.

### Tongue image labeling for analysis

Photographs of each dyed tongue were downloaded, labeled with the participant’s code, and stored on laboratory computers. To facilitate fungiform papillae counting, the line tool in Photoshop CC was used to apply a 4 cm^2^ grid over the anterior tongue region; the 10 mm filter strip served as a length reference. The grid was divided into sixteen 1 cm^2^ regions which correlated to the Subarrays of the mouthpiece’s electrode array and the 4 anterior to posterior positions of the array during testing (Locations) Fig 1. This allowed comparison of fungiform papillae density to electrotactile perception data. In addition, smaller regions of the tongue could be analyzed and compared to identify differences between anterior and more posterior regions and between left and right regions. Files of each photograph with its applied grid were saved as a .jpeg file in the appropriate, coded folder on laboratory computers.

### Fungiform papillae counting

Using images of a dyed tongue with its overlying grid, fungiform papillae were counted using a modified version of the Denver Papillae Protocol [75]. This involved using open-source ImageJ software, version 1.50e Java 1.8.0_73 (64 bit), with a Cell Counter plugin. Two researchers counted each participant’s FP separately, and these counts were entered into a prepared Excel spreadsheet. If the two counts within each 1 cm^2^ region (Fig 1) were within a 10% error, the counts were averaged for a final count. If the difference between the two counts was larger, investigators re-evaluated individual papillae relative to the criteria needed to confirm FP status until counts were within 10%.

### Propylthiouracil taste test

PROP testing strips were prepared using 8.5 g of 6-n-propylthiouracil (Sigma Catalog #P3755) in 500 mls of distilled water. This mixture was stirred on a hotplate until dissolved. Whatman’s No. 1 filter paper was then briefly and completely submerged into the solution, removed and allowed to dry. Dry PROP papers were cut into 1 cm^2^ strips and stored at room temperature in a sterile petri dish sealed with Parafilm. For each experiment, subjects were first asked to rinse their mouths with distilled water. The investigator then removed a single PROP testing strip from the petri dish using sterile forceps and placed it on the subject’s palm. The subject placed the PROP strip onto the anterior tip of their tongue for 15 seconds and rated the intensity of the taste using a ratio scale, the general Labeled Magnitude Scale, which was developed for sensory testing including PROP bitterness perception and comparison. This scale ranges from 0 (not detectable) to 100 (strongest imaginable sensation of any kind) [77, 78]. Participant responses were recorded by hand and then included in each subject’s coded file.

### Data analysis and statistics

Data obtained from electrotactile stimulation and tongue staining of the anterior and medial 4 cm^2^ of the tongue were used for analyses. This area corresponds to Location 1 (L1, most anterior 1 cm of tongue), Subarrays 2 (S2, left) and 3 (S3, right) and Location 2 (L2), Subarrays 2 and 3 (Fig 1). Analyses were restricted to this area for multiple reasons. With respect to electrotactile stimulation, we previously demonstrated that these 4 regions are most sensitive to ETS. Participants chose an initial ETS setting based on stimulation of this area and eliminating more posterior and lateral regions increased the probability that active electrodes at a constant setting would be perceived in each region analyzed. As shown previously, regions posterior to the first anterior 2 centimeters are less sensitive to ETS and stimulation may not be perceived. In addition, the electrode array was more likely to be fully in contact with the tongue in the chosen regions. With respect to fungiform papillae analysis, FP counts were more straightforward in the anterior-medial area since lateral curved tongue areas were avoided. Photographing stained tongues for analysis is difficult and anterior-medial tongue regions were more likely to have even staining and be in sharp focus in images, facilitating accurate counting of fungiform papillae [35]. Furthermore, although our ETS stimulation protocol included random stimulations with only one electrode activated, only data collected when two electrodes were active were included in analyses.

To visually represent each subject’s perception of lingual electrotactile stimulation, data from individual experiments were imported into Excel and MatLab for graphical analysis. Discrimination ability for a region was assessed by determining the minimum distance between electrodes for which the subject reported feeling two distinct stimuli. For plotting, the locus on an array or graphic, this minimum discrimination distance was plotted at the center point of the two discreet stimuli. Distances tested were 2, 4, 6, and 8 mm between electrodes. Tongue graphs reflect the minimum 2-point discrimination distance for a specific locus. Data from both perceived intensity and two-point discrimination ability were interpolated using array and matrix tools in MatLab and the ‘inpaint nans’ function using the spring metaphor method between tested loci to create maps of the tongue showing the distribution of ETS perception for each participant. For analysis of two-point discrimination ability, data from regions where the subject could not correctly discriminate electrodes spaced 8 mm apart were extrapolated/assumed to be 10 mm.

For statistical analysis, factors and their levels were defined as Location (1,2), Subarray (2,3), Orientation of electrodes (V = anterior-to-posterior, H = left-to-right) and Distance (2, 4, 6, 8 mm). Additionally, two numerical predictors were defined: Taste (PROP sensitivity) and Papillae (papillae density). For both the perceived intensity of stimulation (PerInt) and the perceived number of active electrodes (PerNum), subjects reported observations as Likert values, which corresponded to [1, 2, …, 10] and [0, 1, 2, 3] respectively. We computed the means of three subsample observations, holding the levels of Subject, Location, Subarray and Distance fixed and used these as our response variable for data analysis. There were 15 subjects, yielding a total of 1440 observations.

Implementation of exploratory data analysis resulted in the generation of summary statistics and graphics. A linear mixed model was fit to both responses where the factors (Location, Subarray Orientation and Distance) were fixed effects. Random effects were included in the model by subject and subject interaction with Location and with Subarray. Additionally, subject-specific random slopes with respect to papillae density were included. All random effects were assumed to be independent and normally distributed with expectation 0 and some variance component. For each subject’s orientation level (V, H) at a specific location on the tongue, the four distances (2, 4, 6, and 8 mm) were considered to be a cluster in order to model spatial correlation. Two models were initially compared–a “reduced” model containing main effects and a “full” model containing all possible two-way interaction between the 6 explanatory variables. Using maximum likelihood, a likelihood ratio test was done to select the model. The corrected Akaike Information Criterion (AICc) was used to model the covariance structure of the errors among levels of distance. Using restricted maximum likelihood and Kenward-Roger degrees of freedom, we generated F-tests for the fixed effects. Likelihood ratio tests of covariance parameters were also done based on a mixture of chi-squared distributions. Standard residual diagnostic plots were used to check the assumptions of the model. All data analysis was done using SAS for Windows software, Version 9.4.

## Results

### Perceived intensity and discrimination ability for electrotactile stimulation

**Fig 2:**
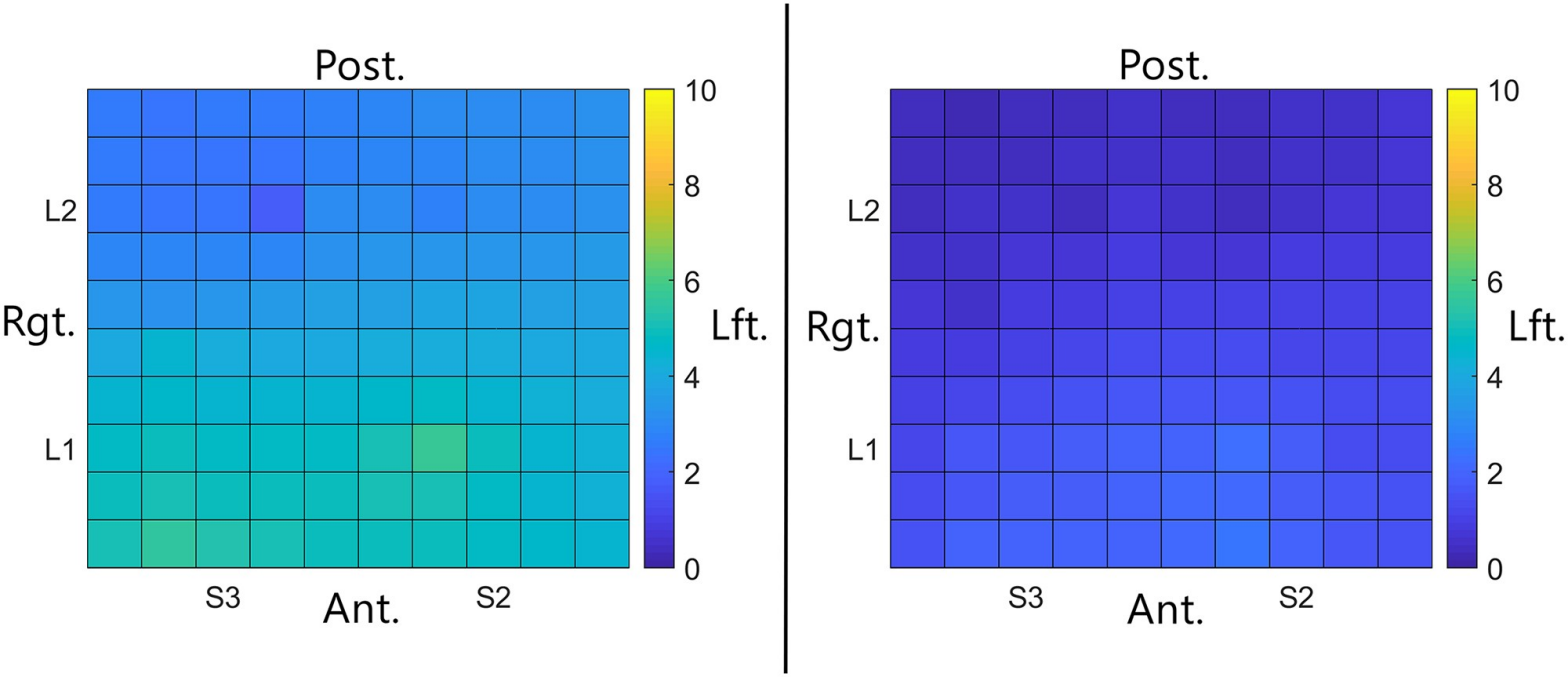
Tongue maps demonstrating average reported perceived intensity for the tested region of two subjects. The left map shows results from a participant with higher overall perceived intensity for ETS, with highest intensity reported for electrodes in anterior medial regions (Location 1, Subarrays 2 and 3). The right map shows results from a participant with lower perceived intensity across the tested area as indicated by the darker colors. The scale to the right of each map reflects the intensity represented by color and the orientation of the map is indicated by the labels surrounding each map. Note that for both participants, stimulation of the anterior region resulted in higher perceived intensity than stimulation of the posterior region.

**Fig 3:**
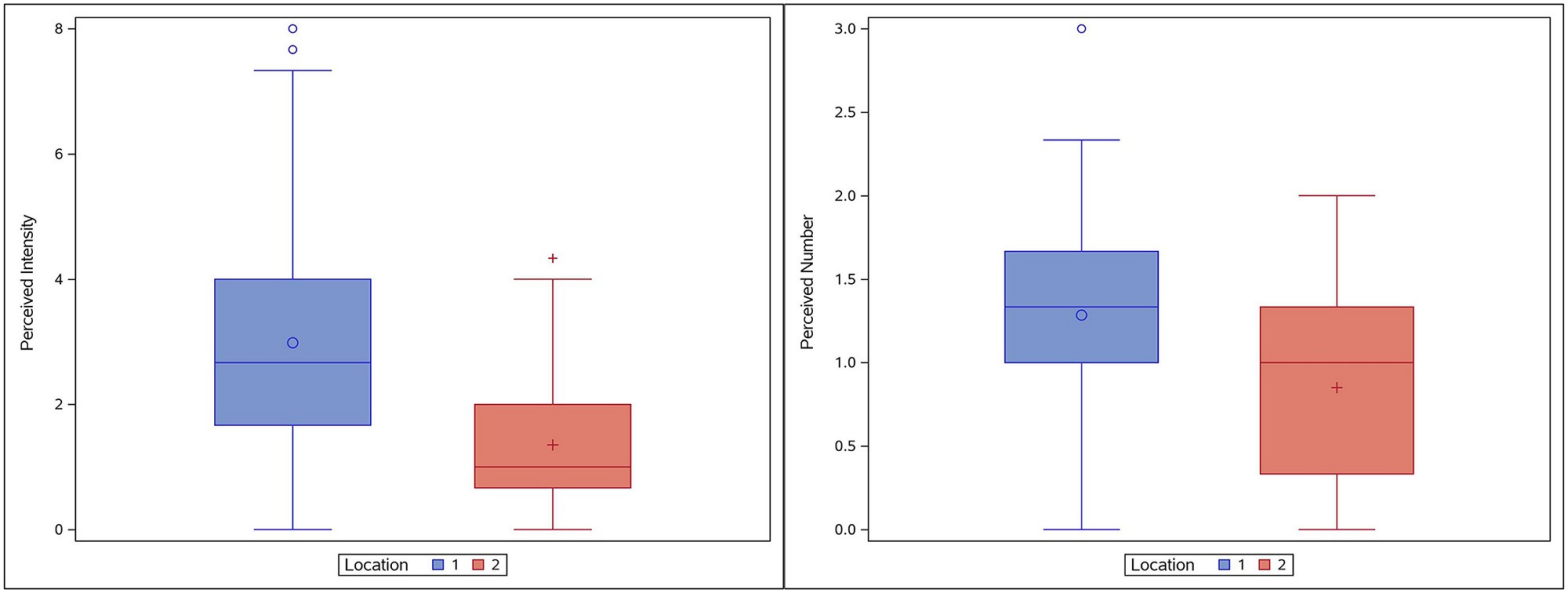
Box plots for perceived intensity and discrimination ability. Left: average perceived intensity was significantly higher in Location 1 relative to Location 2. Right: average discrimination ability was only slightly better in Location 1 as measured by the average number of electrodes which were perceived across all participants. Better discrimination ability is indicated when the average is close to two since only data collected while two electrodes were active were used in this analysis.

Similar to previous studies [35], perception of lingual electrotactile stimulation (ETS) varied substantially between participants and between the 4 tested tongue regions (Fig 2). The perceived intensity of active electrodes was high for some participants (Fig 2, left panel), whereas others reported lower perceived intensities (Fig 2, right panel). However, in general, higher perceived intensities were reported for the anterior tongue region (Location 1) relative to the posterior region (Location 2) (Fig 3).

When considering all participants, for Location 1, the estimated mean intensity was 2.9222 (SE = 0.2974) while for Location 2, the estimated mean intensity was 1.5026 (SE = 0.3137). The 95% confidence interval for the difference between the two location means (1 & 2) was (0.6702, 2.1692). Thus, despite the wide variability between participants, mean perceived intensity for ETS was higher in Location 1, consistent with previous studies.

**Fig 4:**
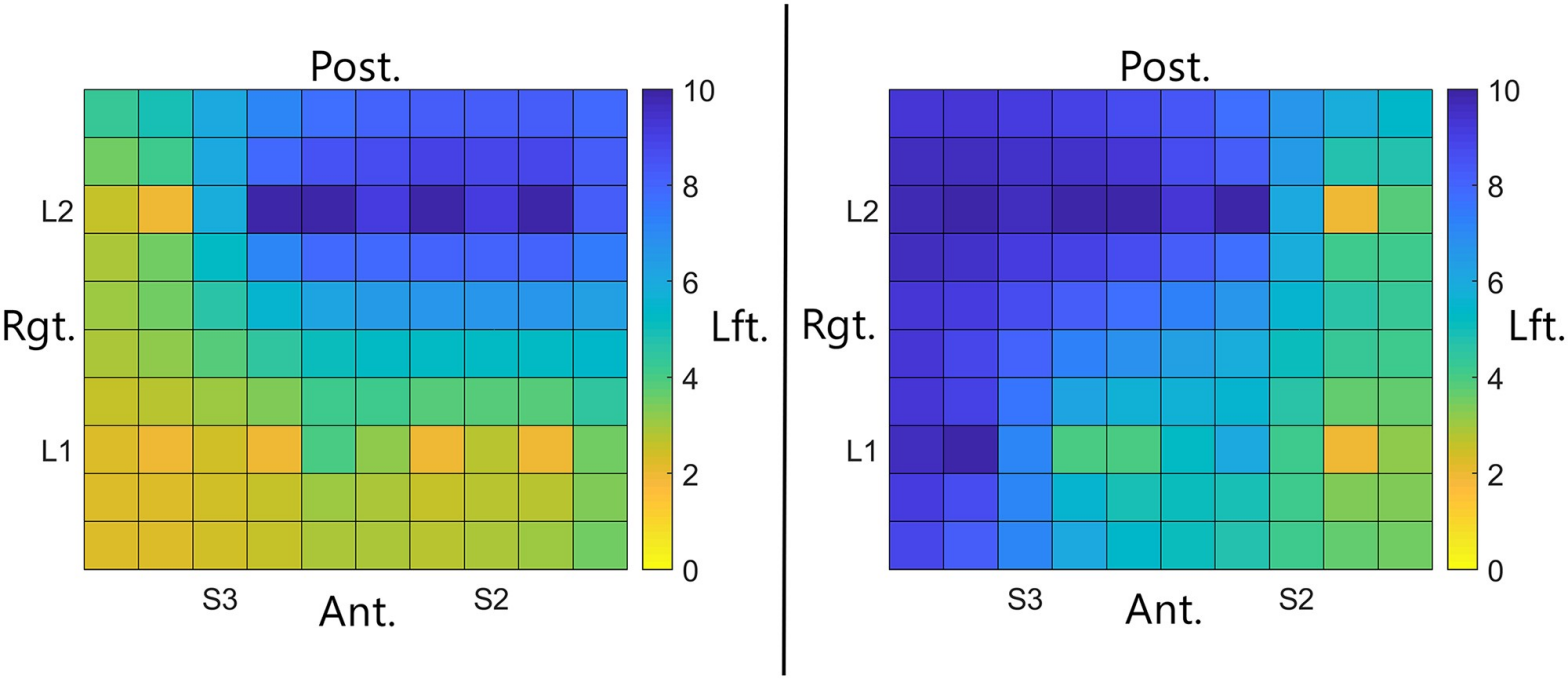
Tongue maps demonstrating the minimum discrimination distance for the tested region of two different subjects. For these maps, orange indicates that the subject was able to discriminate two active electrodes that were 2 mm apart whereas blue indicates the participant was able to discriminate electrodes that were 6–10 mm apart. The left map shows results from a participant who was able to discriminate closely spaced electrodes on the anterior centimeter (L1) and on the right side of the tongue (S3). The right map shows results from a participant who had less discriminatory ability overall as indicated by more blue in the map. This participant had the best discrimination on the left side of the tongue (S2). These two maps illustrate the extreme variability between some individuals with respect to discrimination ability in the anterior medial tongue (L1 & L2, S2 & S3).

The ability to discriminate between active electrodes also varied between participants and between tongue regions as reported previously (Fig 4) [76]. In general, discrimination ability was better in the most anterior 1 cm of the tongue (Location 1) as indicated in the left panel of Fig 4. More participants were able to discriminate electrodes that were 2 mm apart in this region. In contrast, in Location 2, discrimination ability was generally limited to electrodes that were spaced farther apart. However, on average, the ability to perceive discrete stimuli was not significantly different between Locations 1 and 2, using an unadjusted alpha = 0.05 (P-value = 0.0777, Fig 3 right panel).

### Fungiform papillae

**Fig 5:**
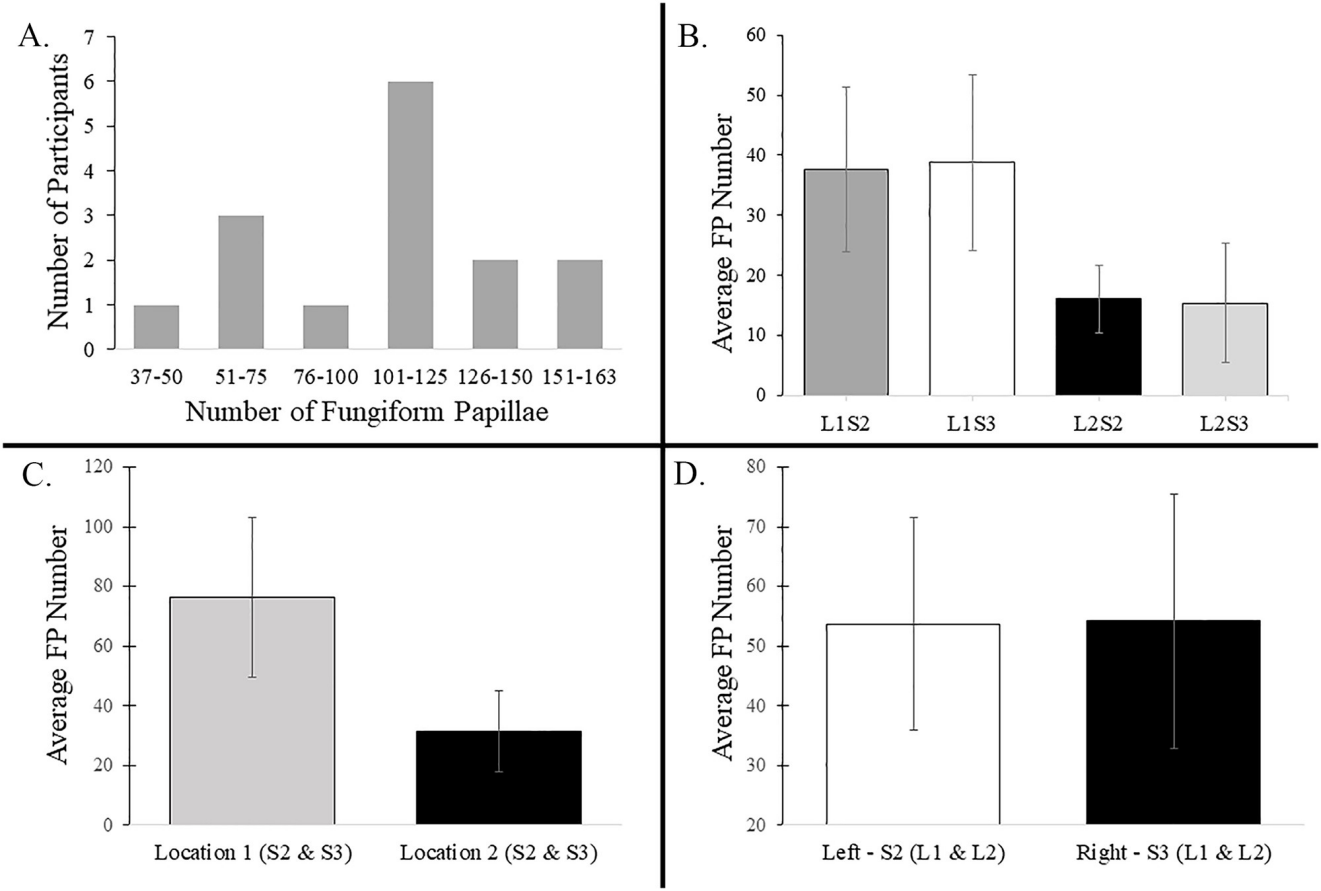
Fungiform papillae (FP) counts from all participants. A. The total number of FP in all 4 regions varied among the participants. Eighty percent of participants had between 51–150 papillae and 40% of participants had between 101–125 papillae. B. Average FP number in each Subarray. The most medial two subarrays in location 1 (L1S2 and L1S3) had the highest average number of papillae in the participant pool. C. Average FP number in location 1 was higher than location 2. D. The average FP number for Subarray 2 (left) and Subarray 3 (right) was 54 and 55 respectively, and not statistically significant.

Fungiform papillae (FP) density varied between individuals and between the different tongue regions as expected [64, 79]. The total number of FP in the tested area for individual participants (Locations 1 and 2, Subarrays 2 and 3) ranged from 37–163, providing a wide range of papillae numbers in subjects for analyses (Fig 5A). The majority of participants (80%) had between 51–150 fungiform papillae and 40% had between 101–125 papillae. The mean number of papillae for all participants was 108. All 15 participants had more papillae in Location 1 (range: 28–117 for Subarrays 2 and 3) than in Location 2 (range; 9–66 for Subarrays 2 and 3) and in most cases (87%, 13/15) individual participants had more than twice as many FP in Location 1 (Subarrays 2–3) compared to Location 2 (Fig 5B). The mean papillae number in Location 1 was 76.4, SD = 25.75, and in Location 2 was 31.53, SD = 13.1.

On average, participants had the same number of FP on the left as the right. Consistent with this, 6 participants had papillae numbers that differed by 10% or less when comparing the right to the left side. However, for 9 individuals, there was more than a 10% difference in papillae number between the left and right, for 7 participants, the difference was more than 20% and for 3 participants, the difference in lingual papillae in the left and right regions differed by more than 30% Table 1). For participants that had more than 10% difference in papillae number between the two sides, 5 had more papillae on the right, and 4 had more papillae on the left. The diversity of FP number between participants and tongue regions thus allowed us to ask whether differences in perceived intensity and discrimination ability is correlated with differences in fungiform papillae density.

**Table 1:** Numbers of papillae for individual subjects to the left of the midline (S2) compared to the right of the midline (S3) for Locations 1 and 2 combined.

	Subject #	S2	S3	Difference
1	6	42	23	45%
2	1	26	42	38%
3	7	57	88	35%
4	4	68	50	26%
5	8	70	93	25%
6	3	37	47	21%
7	9	66	52	21%
8	8	55	64	14%
9	5	55	49	11%
10	10	80	72	10%
11	12	40	36	10%
12	14	73	67	8%
13	15	18	19	5%
14	11	58	60	3%
15	13	61	61	0%

Note that 9 subjects had fungiform counts that differed more than 10% when comparing right and left sides, and Subjects 6, 1 and 7 had substantially different numbers of FP on each side of the tongue (greater than 30% difference). For some individuals, there were more papillae on the right, but others had more papillae on the left. Also note that 6 Subjects (#10–15) had roughly the same number of papillae on each side of the tongue (less than or equal to 10% difference).

### Propylthiouracil

Propylthiouracil is a bitter compound that is perceived by some people, but not by others and although the ability to perceive PROP is determined by the amino acid composition of a protein receptor on type II taste cells, there is some evidence that PROP tasters are more sensitive to somatosensory stimuli [63]. Thus, we tested the ability of our participants to taste PROP to determine whether this characteristic is associated with ETS perception. Participants were instructed to sample a previously prepared piece of PROP paper by placing it on the anterior tip of the tongue, then rate PROP intensity using the gLMS scale [77]. Total responses from our participant pool ranged from 0–80, where 0 represented a complete lack of taste and 100 represented the strongest imaginable sensation of any kind. Based on previous studies, these responses were used to categorize individuals into 3 PROP classes: non-tasters (below 23), medium tasters (23–49) and super tasters (above 50) [80–83]. Using these parameters, our participant pool consisted of 7 non-tasters, 3 medium tasters, and 5 supertasters. These data could also be simplified as 7 non-tasters and 8 tasters, thus we had a diverse population which allowed comparisons between PROP taster status and ETS perception.

### ETS relative to fungiform papillae density

Since our participants varied with respect to ETS perception and FP number and distribution, we were able to compare these characteristics to determine whether the perception of active electrodes was associated with fungiform papillae.

**Fig 6:**
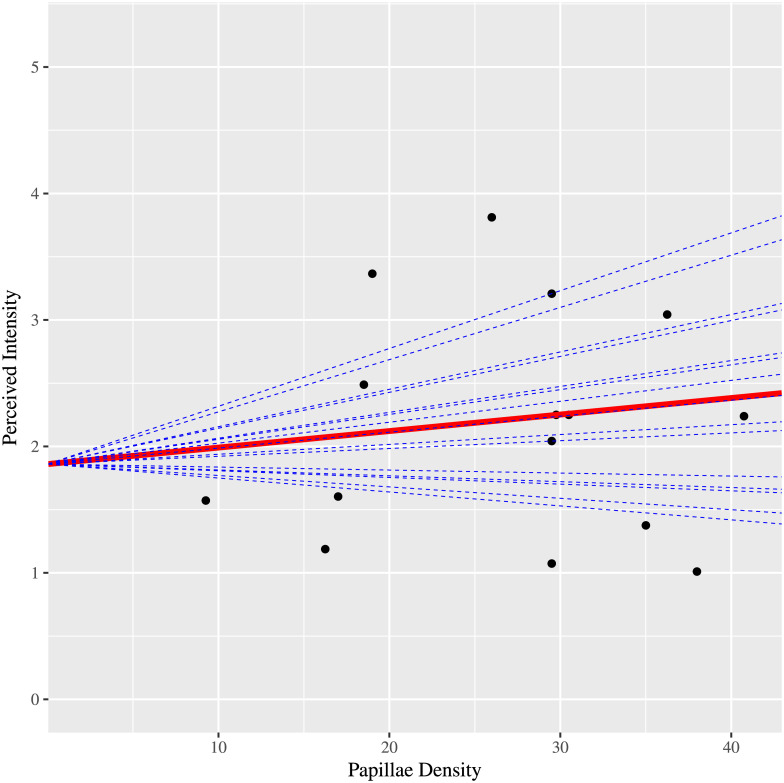
Relationship between papillae density and perceived intensity. Each dashed line represents the conditional subject-specific profile calculated by incorporating the best linear unbiased predictions of all the random effects for each subject. The red line is the marginal line from the fitted linear mixed model for a typical, average subject; that is nominal Taste (31.33) averaged over Location, Subarray, Distance and Orient. The 15 black dots are the observed means (n = 32) for each subject. Note that the red line is quite flat which indicates that on average, there is no association between papillae density and perceived intensity (P-value = 0.3759).

We first addressed the question of whether perceived intensity of ETS related to fungiform papillae density. Other fixed effects were also examined. Results from the likelihood ratio test indicated to use the simpler, main effects model (P-value = 0. 4624). Based on AICc, an autoregressive order-1 error covariance structure was selected to model mild correlation of the errors among levels of distance (P-value = 0.0169). Consistent with previous results [35], perceived intensity was associated with the location of stimulation (Table 2, P-value = 0.0005). As mentioned, active electrodes presented to the most anterior 1 cm of the tongue resulted in the highest perceived intensity. In contrast, the subarray, distance between electrodes, and orientation of electrodes were not associated with perceived intensity in this study. Similarly, on average, perceived intensity of ETS was not associated with differences in papillae density for different participants (Fig 6, Table 2, P-value = 0.3759).

**Table 2:** Tests of fixed effects for perceived intensity of ETS.

Type 3 Tests of Fixed Effects				
Effect	Num DF	Den DF	F Value	Pr > F
**PROP Taste**	1	12.3	0.02	0.8938
**Location**	1	30.3	14.95	0.0005
**Subarray**	1	13.8	0.46	0.5092
**Distance**	3	332	1.19	0.3129
**Orientation**	1	133	2.39	0.1248
**Papillae**	1	21.8	0.82	0.3759

Papillae density was not associated with perceived intensity for electrotactile stimulation (P-value = 0.3759). As demonstrated previously, Location of ETS stimulation was correlated with perceived intensity for active electrodes (P-value = 0.0005).

We found insufficient evidence that the variance component for subject random intercepts was greater than 0 (P-value = 0.3195). However, the variance component associated with subject-specific random Papillae slopes was greater than 0 (P-value = 0.0262). This means that each subject imparted their own conditional effect on the overall behavior of papillae density with respect to perceived intensity. As mentioned in the methods section, to address smaller/larger Subarray variation depending on the Subject, we included a random effect associated with Subarray (2 vs. 3) for each Subject. The variance component for these random Subarray effects was significantly greater than 0 (P-value < 0.0001). We observe the same behavior with respect to Location (P-value < 0.0001).

**Fig 7:**
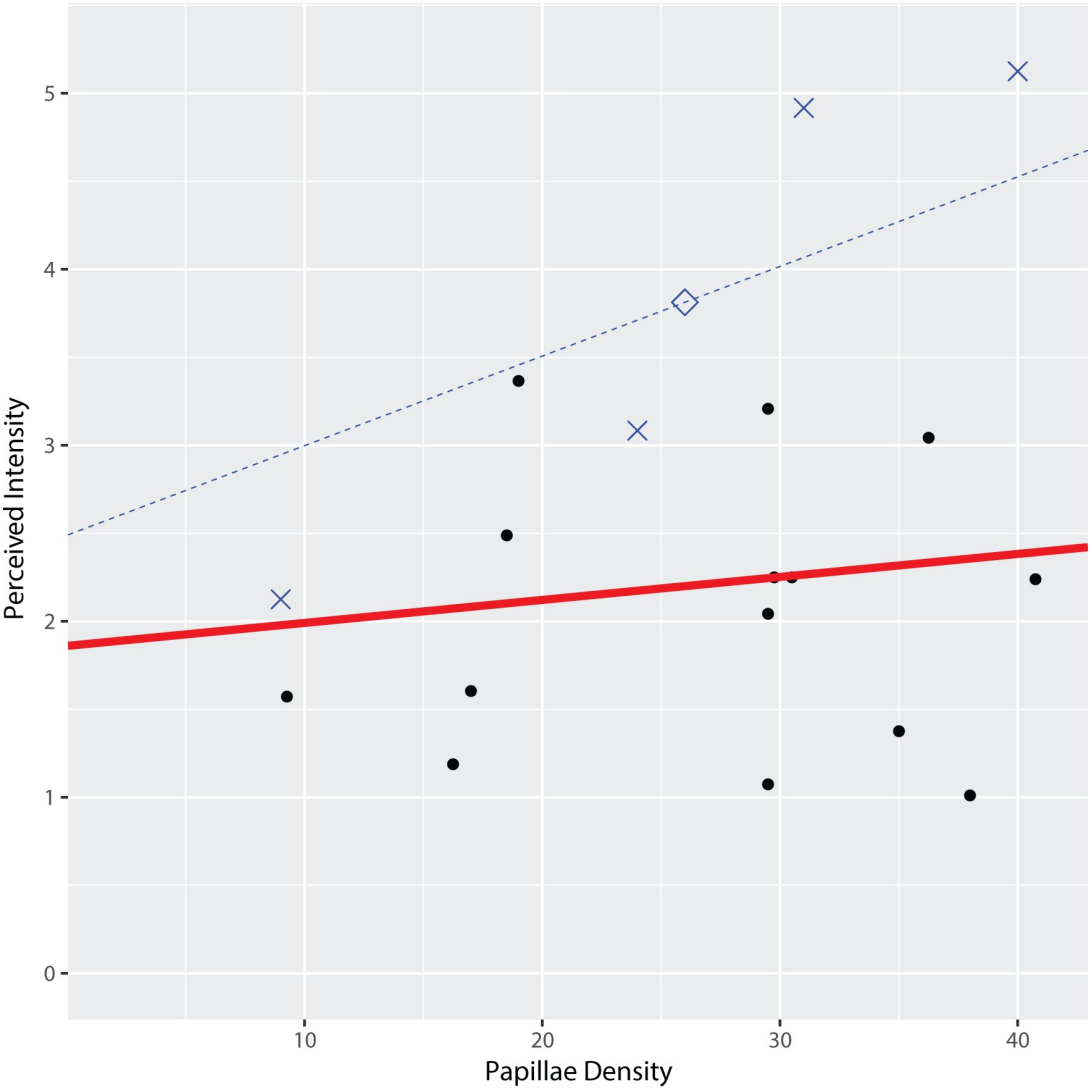
Relationship between perceived intensity and fungiform papillae density for one participant relative to the average relationship in the set of participants. The dashed blue line indicates the conditional subject-specific profile calculated by incorporating the best linear unbiased predictions of the random effects for this subject. The red line is the marginal line from the fitted linear mixed model for a typical, average subject as shown in Fig 6 and the black dots indicate the observed means. Blue Xs are the raw observed perceived intensity means at each Location-by-Subarray region of the tongue (n = 8) for one subject, while the blue diamond is their overall mean. The blue dashed line is a conditional line averaged over Location, Subarray, Distance and Orientation obtained from the fitted model. Notably, we found evidence of a (positive) linear association between papillae (Pap) and perceived intensity (PerInt, P value = 0.0361) for this participant.

Interestingly, calculation of the conditional subject-specific profiles by incorporating the best linear unbiased predictions of the random effects for each subject revealed that data from at least one participant showed a possible association between papillae density and perceived intensity (Fig 6). Further analysis of one of these subject’s 32 observations by fitting a reduced linear mixed model only for this subset of data, revealed evidence of a positive linear association between papillae density and perceived intensity of ETS for this participant (Fig 7, P-value = 0.0361).

**Fig 8:**
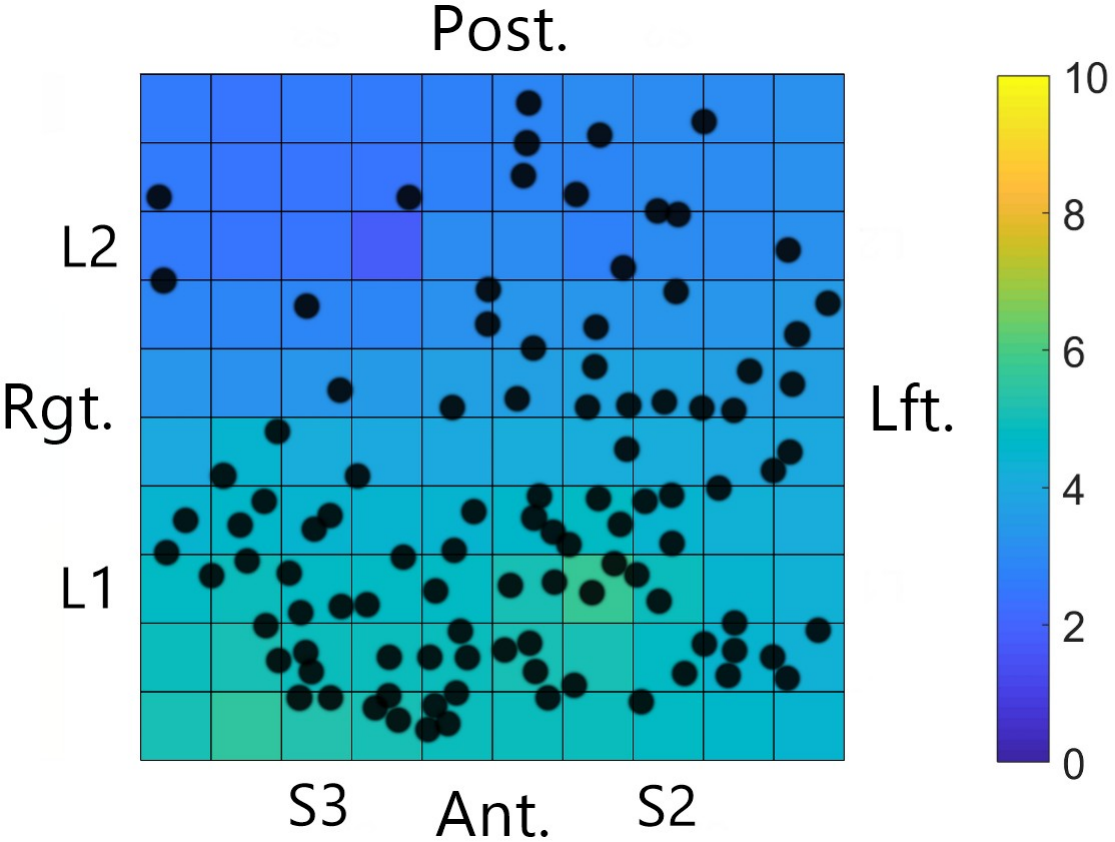
Overlay of perceived intensity map and fungiform papillae for the participant that demonstrated a statistically significant positive linear association between papillae and perceived intensity. Colors represent perceived intensity (PerInt), with lighter blue and green indicating higher PerInt as indicated by the scale to the right of the tongue map. Black dots represent individual fungiform papillae (FP) for this participant in the related regions. Note that there are more FP in the anterior region where there is also higher perceived intensity.

The positive association between papillae and perceived intensity for electrotactile stimulation was investigated further by combining the participant’s perceived intensity heat map with a map of the fungiform papillae in the tested region. More FP were present in regions where the participant reported higher intensities (Fig 8).

**Fig 9:**
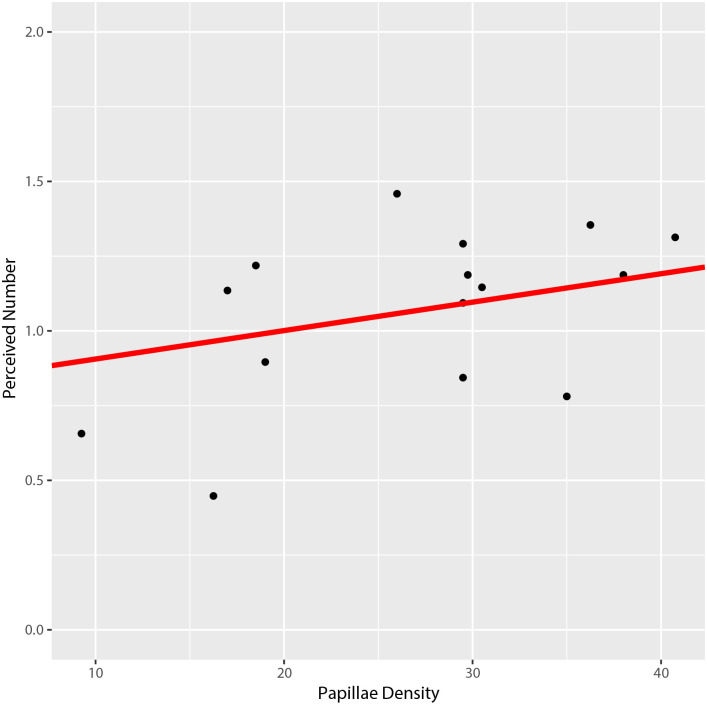
Relationship between papillae density and perceived number of active electrodes. The red line is the marginal line from the fitted linear mixed model for a typical, average subject. The 15 black dots are the observed means (n = 32) for each subject. Note that the red line has a positive slope, which shows that there is an association between papillae density and perceived number of electrodes (P-value = 0.0203).

We next tested whether fungiform papillae (FP) density is related to the ability to discriminate between electrodes. For this analysis, results from the likelihood ratio test indicated that the simpler, main effects model should be used (P-value = 0.8113). Based on AICc, a compound symmetric error covariance structure was selected to model very mild correlation of the errors among levels of distance (P-value = 0.0568). The results indicated that participants with more FP were better able to discriminate active electrodes, relative to participants with fewer FP (Table 3 Fig 9, P-value = 0.0203).

**Table 3:** Type 3 tests of fixed effects for perceived number of electrodes.

Type 3 Tests of Fixed Effects				
Effect	Num DF	Den DF	F Value	Pr > F
**PROP Taste**	1	12.6	0.71	0.4160
**Location**	1	35	3.30	0.0777
**Subarray**	1	14	1.74	0.2087
**Distance**	3	357	0.44	0.7237
**Orientation**	1	73.6	0.17	0.6848
**Papillae**	1	75.8	5.62	0.0203

Papillae density correlates with ETS discrimination. Using an unadjusted alpha = 0.05, papillae density is significant (P value = 0.0203), while location was, perhaps, marginally significant (P value = 0.0777).

Examining the perceived number covariance parameters, we found insufficient evidence that the variance component for subject random intercepts was greater than 0 (P-value = 0.1504). Furthermore, the variance component associated with subject-specific random papillae slopes was estimated to be 0, indicating that there were no individual differences in the overall behavior of papillae density with respect to perceived number of active electrodes (note the absence of conditional subject-specific profile lines in Fig 9). However, subarray variation appeared to marginally depend on the Subject (P-value = 0.0663) and there was similar, stronger behavior with respect to Location (P-value = 0.0039).

### ETS relative to ability to perceive 6-n-propylthiouracil

Finally, we tested whether PROP tasting ability was associated with ETS perception. Our results indicate that PROP taster status is unrelated to perceived intensity for ETS (P-value = 0.8938), and also unrelated to ability to discriminate active electrodes (P-value = 0.4160, Tables 2 and 3).

## Discussion

In the experiments described here, we tested whether fungiform papillae (FP) density and distribution or the ability to detect 6-propylthiouracil (PROP) are associated with perception and discrimination ability of electrotactile stimuli on the tongue. The ultimate goal of our research is to determine how to better design lingual electrode arrays for sensory substitution. Our results indicate that on average, the numbers of fungiform papillae do not positively or negatively correlate with perceived intensity of active electrodes across the tongue surface. However, we did find evidence of a statistically significant correlation for at least one individual. For this subject, tongue regions with higher FP density were associated with higher perceived intensity of ETS, suggesting that more research is needed. The results presented here indicate that tongue regions with more FP are associated with better ability to discriminate two active electrodes. This suggests that individuals with more FP may be better able to use lingual ETS for sensory substitution. Furthermore, electrode arrays should be designed such that active electrodes stimulate regions with more fungiform papillae, which for most people includes Location 1, Subarrays 2 and 3 (the 2 centimeter region flanking the midline of the tongue in the most anterior centimeter). For some individuals, this placement may also result in increased perceived intensity for ETS. Finally, the ability to detect the bitter compound 6-n-propylthiouracil did not correlate with either perception of ETS intensity or discrimination ability in the present study.

Our findings are consistent with, and extend, those done previously. Similar to previous work, we found enhanced sensitivity (increased perceived intensity) and increased discrimination ability in the anterior tongue in response to lingual ETS [35, 72]. Fungiform papillae density and distribution varied within and among participants as reported previously, and PROP sensitivity varied among participants as expected. The increased discrimination in subjects with more FP suggests that mechanosensitive somatosensory fibers activated by ETS may be concentrated in fungiform papillae. Like the epidermis, the lingual epithelium contains multiple types of mechanoreceptors including low (LTMR) and high threshold receptors (HTMR). The former responds to non-painful stimuli and the latter responds to painful stimuli. The anatomical distribution of LTMR fibers in specific tongue regions innervated by the lingual nerve of the tongue has been carefully studied using microneurography and calibrated filaments [32]. These studies indicated that superficial (LTMRs) can be classified as slowly or rapidly adapting based on responses to a sustained stimulus. Both receptor types can have small receptive fields (range 1–19.6 mm^2^) that are circular or oval in shape and are concentrated in anterior tongue regions [32]. The size of a receptive field contributes to discrimination ability since separate stimulation points are discernable only if they interact with receptive fields of two different neurons. It should be noted that receptive field characteristics determined using mechanical stimuli are likely dependent upon properties of the surrounding tissue, associated peripheral cells or structures, or the distribution of neuronal projections, whereas receptive fields related to electrical stimuli may be influenced by the electrical resistance of the surrounding tissue and electrical properties of the electrode-tongue interface. Animal studies indicate that somatosensory fibers can be found in both fungiform papillae, which contain taste buds, and in the surrounding filiform papillae, which do not [60, 84, 85]. Some neuroanatomical studies suggest that somatosensory fibers are concentrated in fungiform papillae [61, 86]. Our result showing that regions with more FP are associated with a better ability to discriminate ETS, are consistent with the idea that rapidly adapting low-threshold mechanoreceptors (RA LTMR) in particular may be concentrated in FP.

Our results suggest that lingual ETS for applications involving transmitting information via the mechanoreceptors of the tongue should be designed based on the fungiform papillae density for maximum effectiveness. Arrays or stimuli location should be designed so that electrodes contact regions with more fungiform papillae, which for the average individual is the 2 cm region at the most anterior medial part of the tongue. Any application using lingual ETS will be enhanced by designing a standard array based on our findings. Eventual training protocols may also need to consider FP distribution and density. We found that there is subject-dependent variation between subarrays with respect to ETS perception and discrimination. Fungiform papillae distribution and number can vary from subject-to-subject and between subarrays within a subject; our papillae counts revealed that 9 out of 15 participants had FP numbers that differed by more than 10% when comparing the left and right (subarray 2 vs 3). For example, in Location 1, subject 7 had 57 papillae in S2 and 88 papillae in S3 (Table 1). Further experiments will be designed to determine if FP asymmetry influences ETS pattern perception. Specifically, it would be interesting to determine if subject 7 is better able to perceive specific patterns on the right side of the tongue. Importantly, the first step in this process would be to simply assess the distribution of fungiform papillae, which can be done using diluted food dye and a digital camera.

Some individuals have more FP overall than others and this might allow them to more easily discern different electrotactile patterns that could be associated with specific sensory information. In contrast, people with fewer FP may need more training to learn to discriminate ETS patterns. Our results with lingual ETS are consistent with studies using a somatosensory-based letter-recognition task that demonstrated a correlation between enhanced tactile discrimination and FP density [66], but more research is needed. Another caveat to consider is that discriminating between active electrodes is a unique task and although many individuals have experienced a similar sensation when drinking carbonated beverages, the perception of individual buzzing sensations from ETS is typically a new experience. To effectively report discrimination of such stimuli, study participants may need more instruction, training and time prior to testing. Additionally, our participant pool was limited to 15 individuals, and increasing the number of subjects may reveal further details about the relationship between FP density and discrimination ability.

Investigating the relationship between fungiform papillae and the perceived intensity of electrotactile stimuli also requires further study since we discovered at least one participant with a significant correlation between the two variables. Perceived intensity of ETS is difficult to study since perception varies between individuals and is difficult to assess and standardize. Comparing ETS sensations to calibrated filaments may be beneficial and enable subjects to have a frame of reference for intensity ratings. Increased experience with the ETS prior to testing may also improve accuracy of participant responses. In addition, we may need to extend the range of our available initial “intensity” settings for individual subjects by adjusting parameters. Each participant chose an initial setting after trying a fixed range of stimulus options. These settings were controlled by varying pulse width, and the number of bursts of pulses in a specified interval as described previously [35]. Expanding this range and selecting different settings for Location 1 and Location 2 may increase perception of the electrotactile stimuli especially in Location 2. A potential additional source of error was the firmware used on the experimental equipment, which allowed for a range of delays between pulses on “simultaneously” active stimuli [35]. Pulse magnitude could also be adjusted to activate fibers that may be located deeper in the lingual tissue [72]. Another possibility is that our protocol may be too long and subject fatigue may be playing a role, leading to decreased attention to the lingual ETS. The procedure was designed to be comprehensive and 116 different stimulations were presented randomly across each 1 cm strip of tongue during testing. Although this provided us with substantial data, we may be able to reduce the stimuli and time for each testing session. Finally, differences in papillae diameter were found in previous studies to be correlated with lingual spatial resolution [65], and may contribute to perceived intensity variability. Larger papillae may contain a greater number of receptors activated by a single stimulus. Because our data and analyses indicated that there was a relationship between FP density and perceived intensity for at least one subject, differences in perceived intensity may be subtle and require a larger participant pool and improved experimental methods to detect. Future studies will focus on this by seeing if the duration of the stimuli is a factor. Activating two electrodes at a time may have also obscured our results as two stimuli spaced closer together could be perceived as one, more intense stimulus. Testing perceived intensity separately with individual active electrodes may clarify results and will be a goal for future studies.

Finally, it is important to note that the graphical representations of ‘minimum discrimination’ ability would not detect if a subject correctly discriminated two electrodes at a small center-to-center distance, but was unable to discriminate electrodes centered at the original location that were spaced farther apart. Further studies may be needed to determine if this is an issue.

Our results suggest the ability to detect PROP is unrelated to both tactile acuity and oral sensitivity. Previous studies have shown a correlation between these; however, they looked at chemosensation to irritants such as capsaicin and ethanol [70], and mechanosensation using embossed letter identification [65]. Our group is the first to specifically compare PROP taster status and perception to electrotactile stimuli.

In conclusion, our results suggest that visual analysis of fungiform papillae may be beneficial when designing electrode arrays and ETS patterns for sensory substitution applications, especially if individuals have dramatically different numbers of FP in the left or right side of the tongue. Further, the relationship between FP and perceived intensity and discrimination ability requires further study to determine more subtle relationships between papillae characteristics and ETS parameters.
